# Bullous pemphigoid in infants: characteristics, diagnosis and treatment

**DOI:** 10.1186/s13023-014-0185-6

**Published:** 2014-12-10

**Authors:** Agnes Schwieger-Briel, Cornelia Moellmann, Birgit Mattulat, Franziska Schauer, Dimitra Kiritsi, Enno Schmidt, Cassian Sitaru, Hagen Ott, Johannes S Kern

**Affiliations:** Departments of Dermatology and Pediatrics, Medical Center – University of Freiburg, Freiburg, Germany; Children’s Hospital “Klinik am Eichert”, Goeppingen, Germany; Department of Dermatology, University of Luebeck, Luebeck, Germany; Catholic Children’s Hospital Wilhelmstift, Hamburg, Germany; Department of Dermatology, Medical Center – University of Freiburg, Hauptstrasse 7, Freiburg, 79104 Germany

**Keywords:** Bullous skin disease, Skin blistering, Vaccination

## Abstract

**Background:**

Bullous pemphigoid (BP) in infants is a rare but increasingly reported autoimmune blistering skin disease. Autoantibody reactivity is usually poorly characterized. Current guidelines do not address specific aspects of the infantile form of BP. The objectives of this study are to define clinical and diagnostic characteristics of infantile BP and develop a treatment algorithm.

**Methods:**

Detailed characterization of a current case series of five infants with BP from our departments. Comprehensive analysis of all reported cases (1–12 months) with respect to clinical and laboratory characteristics, treatment and outcome.

**Results:**

In total 81 cases were identified (including our own). The mean age was 4.5 months. Moderately severe and severe disease was seen in 84% of cases. Involvement of hands and feet was present in all cases. Immunofluorescence microscopy was comparable with BP in adults. Where analyzed, the NC16A domain of bullous pemphigoid 180 kDa antigen/collagen XVII (BP180) was identified as the major target antigen. BP180 NC16A ELISA values in our cohort were significantly higher than in a control cohort of 28 newly diagnosed adult patients.

50% of patients were treated with systemic corticosteroids, 20% with a combination of systemic corticosteroids and dapsone or sulfapyridine and 10% with topical corticosteroids alone. 14% of patients needed a combination of multiple immunosuppressants. All but one patient reached remission. Relapses were rare.

**Conclusions:**

Presentation of infantile BP is often severe with blistering of hands and feet present in all cases. Pathogenesis and diagnostic criteria are comparable to adult BP, yet BP180 NC16A ELISA levels seem to be significantly higher in infants. The overall disease outcome is favorable. Based on the results of this study we propose a treatment algorithm for infantile BP.

**Electronic supplementary material:**

The online version of this article (doi:10.1186/s13023-014-0185-6) contains supplementary material, which is available to authorized users.

## Background

Bullous pemphigoid (BP, ORPHA703) is an acquired autoimmune disorder presenting with subepidermal blistering, eosinophilia, and severe itch [1-5]. Its incidence is increasing [6,7] and it mostly affects the elderly; it is considered rare in children [8,9]. The first case of BP in a child was described in 1970 based on immunofluorescence diagnosis [10]; the first case of BP in an infant was described in 1977 [11]. Since then, the number of reported pediatric cases has steadily increased, prompting Nemeth et al. to propose diagnostic criteria for childhood BP [12] which included children and adolescents up to 18 years of age. In 2008, Waisbourd-Zinman et al. noticed different clinical presentations depending on the age of affected children [13]. In a literature review, they showed that the majority of cases of childhood BP occurred in small children under the age of 12 months and that these infants presented with a particular clinical picture. All affected infants had acral involvement with or without generalized blistering. The distribution in later childhood was far less uniform and included a subgroup of children with localized genital BP, a presentation not described in infants. These clinical differences led to the distinction of infantile versus childhood BP [13].

Diagnostic results in infantile and adult BP are similar, but serological tests were not performed systematically in many of the reported cases [13]. The gold standard for diagnosis is direct immunofluorescence microscopy (DIF). However, little information is available on the interpretation of ELISA levels [14], inflammatory markers or blood cell counts in infants. Further knowledge, especially about the relevance of ELISA levels might help to assess disease severity and thus influence the choice of medication or duration of treatment.

Concerning the treatment of infantile BP, first line treatment usually consists of topical or systemic corticosteroids. However, there are no stringent therapeutic criteria and there has been very little discussion on the different options for second line treatment. Furthermore, in clinical consensus guidelines on treatment of BP, there is very little, if any, information on treatment in infants [15-18].

Here, we report the diagnostic results and disease course of five children with infantile BP in our care and a comprehensive analysis of all cases reported in the literature. Based on these data – and taking into account the published guidelines for adults as well as special circumstances of treating small infants – we propose a first treatment algorithm for infantile BP.

## Methods

### Infantile BP cohort and adult BP control cohort

Five infantile BP patients presented at or were referred to our departments. They were included in this study after we obtained parental informed consent for participation and took blood and skin samples for diagnostic and research purposes. As a control, BP180 NC16A ELISA levels of a cohort of 28 adult BP patients that were newly diagnosed in the same time period were determined after informed consent was provided. All investigations were conducted according to the declaration of Helsinki criteria.

### Histopathology, immunofluorescence microscopy, immunoblotting and ELISA

Hematoxylin eosin staining of formalin fixed, paraffin embedded tissue sections was performed using standard methods. DIF and indirect immunofluorescence microscopy (IIF) were performed as previously described [19-21]. FITC labeled antibodies used for DIF were anti human IgG, IgA, IgM and C3c (Dako, Hamburg, Germany) at a dilution of 1:200, 1:50, 1:50 and 1:500 respectively. For IIF on salt-split skin, patient sera were diluted 1:10, secondary antibodies used were FITC labeled anti human IgG and IgA (Dako, Hamburg, Germany) at a dilution of 1:100 and 1:25 respectively. Immunoblotting of normal human keratinocyte extracts with patient sera at a 1:20 dilution and alkaline phosphatase anti human IgG (Sigma-Aldrich, Taufkirchen, Germany) secondary antibody was performed as previously described [20,21]. ELISA kits for the detection of BP180- and bullous pemphigoid 230 kDa antigen (BP230)-specific antibodies (MBL, Nagoya, Japan) were used according to the manufacturer’s protocol with the cut-off at 9 U/ml.

### Statistical analysis

Boxplot descriptive statistics of BP180 NC16A ELISA values were performed using GraphPad Prism software (GraphPad Software, La Jolla, CA).

### Literature search

We searched all retrievable English- and foreign-language medical literature using PubMed, PubMed Central, EMBASE, and Google Scholar databases as well as literature cited in the obtained reports. Relevant information was extracted and reviewed to avoid duplications of reports. We included only infants up to 12 months in our review and excluded cases of neonatal BP.

## Results

### Patient cohort/index case

The clinical and laboratory findings of the five patients in our cohort are presented in Table 1. Patient 1 (index case) showed characteristic infantile BP and was the most severely affected; his treatment proved to be the most challenging. He is therefore presented in more detail. The previously healthy three-month-old boy of Algerian descent presented with a one-week history of small blisters on hands and feet and urticarial plaques on the trunk. Impetigo had been ruled out at a nearby hospital but no diagnosis had been made. He had received one oral vaccination against Rotavirus one month prior. No other vaccinations had been given. Apart from mild eczema, there was no family history of skin disease. Over the course of one week the lesions increased in number and size. The patient was irritable and not feeding well.

**Table 1:** **Clinical and laboratory findings of the patient cohort**

	**Age (months)/Gender**	**Extent of Disease/Hands/Feet (HF)**	**OM**	**DIF/IIF/Immunoblot**	**ELISA**	**WBC (Eos in%) Thrombocytes**	**Treatment**	**Time Until Remission/Relapse/Duration of Treatment**	**Special aspects**
1)	3/M	Generalized	+	DIF: IgG, C3 (BM)	Anti BP180	10.4 × 10^9^/l (10)	a) Prednisolone	Initially rapid response with disease control	Family history of atopy
		HF+		IIF: IgG (BR)	136 U/ml	At relapse:	2 mg/kg/d → 1 mg/kg/d	Relapse within 2 weeks after diagnosis on systemic prednisolone (2 mg/kg) and during respiratory tract infection	Rotavirus vaccine
				IB: 180kD pos.	(norm < 9)	54 × 10^9^/l (52)	b) Dapsone 2 mg/kg/d	Slow response after relapse, need for multiple medications	4 weeks prior
					At relapse:	Tc >1000 × 10^9^/l	c) IVIG 1 g/kg × 3	Response to dapsone after 2.5 weeks	
					Anti BP180		d) MMF (2× 600 mg/m^2^/ d)	Duration of treatment: 8 months	
					189 U/ml				
					Anti BP230 neg.				
2)	3/M	Localized with few disseminated lesions HF+	-	DIF: IgG, C3 (BM)	Anti BP180	16.1 × 10^9^/l (23)	a) Topical Prednicarbate	Good response to topical treatment within days	
				IIF: IgG (BR)	90 U/ml		(mid-potency corticosteroid)	No relapse	
				IB: 180kD pos.	(norm < 9)			Duration of treatment: 4 weeks	
					Anti BP230 neg.				
3)	4/M	Generalized	-	DIF: IgG, C3 (BM)	Anti BP180	23.4 × 10^9^/l (20)	a) Prednisolone	Complete remission within 1 week	Vaccination 4 weeks prior
		HF+		IIF: IgG (BR)	156 U/ml		2 mg/kg/d → 1 mg/kg/d	Weaning of steroids within 3 months	(DPTP, HiB, HepB, Rotavirus)
				IB: 180kD pos.	(norm < 9)		b) Dapsone 1.5 mg/kg/d	No relapse	
					Anti BP230 neg.			Duration of treatment: 6 months	
4)	3/F	Generalized	-	DIF: IgG, C3 (BM)	Anti BP180	25.1 × 10^9^/l (13)	a) Prednisolone	Slow response to prednisolone 1 mg/kg	Rotavirus vaccine
		HF+		IIF: IgG (BR)	125U/ml	Tc 860 × 10^9^/l	2 mg/kg/d → 1 mg/kg/d	Rapid response to oral betamethasone 0.3 mg/kg/d	4 weeks prior
				IB: 180kD pos.	(norm < 9)		b) Systemic betamethasone	No relapse upon glucocorticoid tapering	Arterial hypertension
					Anti BP230 neg.		0.3 mg/kg/d	Complete remission under dapsone 0.5 mg/kg/d	Myocardial hypertrophy
							c) Dapsone	Treatment ongoing	→ Propranolol
							1 mg/kg/d → 0.5 mg/kg/d		
5)	7/M	Generalized	-	DIF: IgG, C3 (BM)	Anti BP180	27.3 × 10^9^/l (9)	a) Prednisolone 1 → 0.5 mg/kg/d	Rapid response to oral betamethasone	
		HF+		IIF: IgG (BR)	154 U/ml	Tc 599 × 10^9^/l	b) Systemic betamethasone	Full remission after 2 months	
				IB: 180kD pos.	(norm < 9)		0.4 mg/kg/d → 0.2 mg/kg/d	No relapse	
					Anti BP230 neg.		c) Dapsone 0.5 mg/kg/d	Treatment ongoing	

**HF:** Hands/Feet + present, − not present; **OM:** Involvement of oral mucosa; + present, − not present; **DIF:** Direct immunofluorescence microscopy; **IIF:** Indirect immunofluorescence microscopy; **IB**: Immunoblot; **BM:** basement membrane; **BR:** Blister roof; **WBC** White blood cell count; **Eos**: eosinophil granulocytes; **Tc:** thrombocytes; **DPTP**: Diphteria, Pertussis, Tetanus, Poliovirus; **HiB:** Haemophilus influenzae type b; **HepB:** Hepatitis B.

Generalized disease = Moderately severe and severe disease.

**Figure 1:**
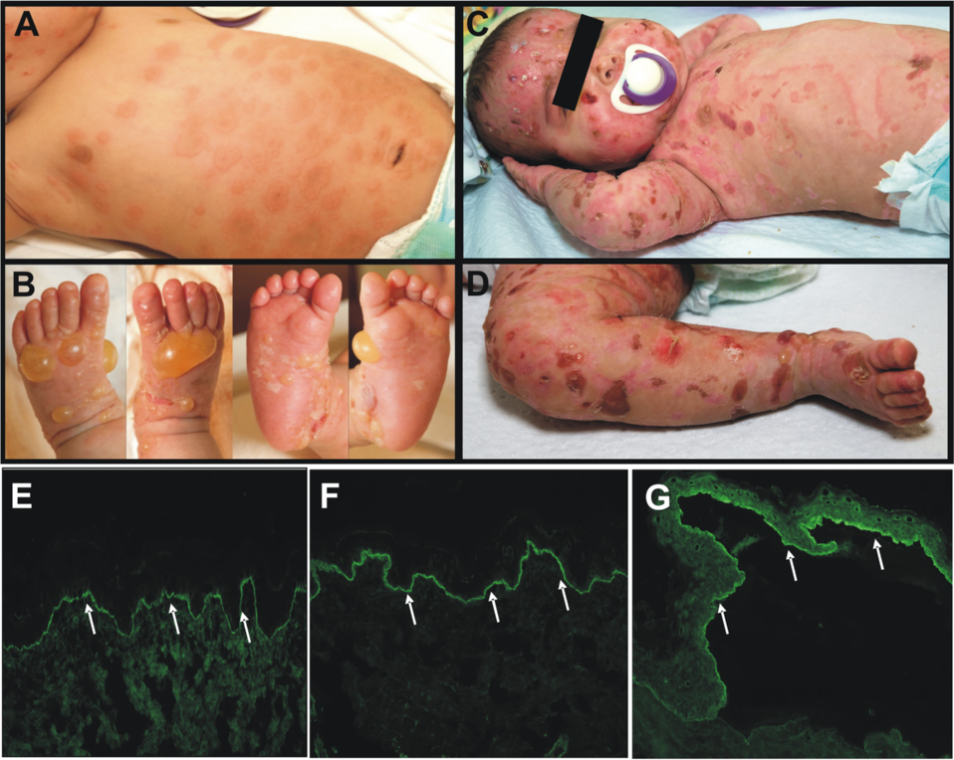
**Clinical and diagnostic hallmarks of infantile BP.** Patient 1 at initial presentation: **A**, urticarial plaques on the trunk. **B**, firm blisters and bullae on the hands and feet. **C**, **D**, Patient 1 after relapse with severe blistering on 2 mg/kg prednisolone daily. Direct immunofluorescence microscopy: **E**, linear IgG and **F**, linear C3c depositions along the basement membrane zone (white arrows, 200× original magnification). **G**, indirect immunofluorescence on salt-split skin reveals circulating IgG antibodies that bind to the blister roof, which is diagnostic for BP (white arrows, 200× original magnification).

On clinical examination, he had firm blisters and bullae predominantly on the hands and feet, as well as urticarial plaques with an elevated rim and a dusky center. These plaques were predominantly located on the trunk but also present on all other areas of the body (Figure 1A, B). The Nikolsky sign was negative; there were no mucosal lesions.

Blister fluid microscopy demonstrated mainly eosinophil granulocytes; cultures from blister fluid remained sterile. Blood testing, including a full blood count, showed normal values with the exception of a peripheral eosinophilia of 10%. Punch biopsies were performed for histologic and immunofluorescence analyses. Histology showed dermal edema and eosinophil inflammatory infiltrate (not shown). DIF revealed linear staining of IgG (Figure 1E) and complement component C3 (Figure 1F) along the basement membrane zone. IIF microscopy showed circulating IgG autoantibodies binding to the epidermal side of the salt-split skin (Figure 1G). ELISA testing with recombinant NC16A domain of BP180 was strongly positive (136 U/ml, norm <9 U/ml). The findings were diagnostic for BP.

Initially, we treated with potent topical corticosteroids and oral antihistamines, which did not lead to significant improvement. After confirmation of the diagnosis, a treatment with prednisolone up to 2 mg/kg/day was initiated. After a brief period of clinical improvement and disease control, the patient had a respiratory tract infection in the course of which he developed severe blistering. At this time he was still on 2 mg/kg prednisolone daily (Figure 1C, D). Peripheral blood count showed leucocytosis with a maximum of 54 G/l (52% eosinophils) and significant reactive thrombocytosis (>1000 G/l) with signs of increased coagulation activity necessitating treatment with acetylsalicylic acid. The ELISA value for BP180-specific antibodies at this point was 189 U/ml. After confirming normal glucose-6-phosphate dehydrogenase levels, we added dapsone at a maximum dose of 2 mg/kg daily, controlling for the development of methemoglobinemia. As the blistering continued to progress, we added intravenous immunoglobulins (IVIG) 1 g/kg three times. Yet the patient developed more cutaneous and additionally intraoral blisters causing refusal of oral intake. He also developed persistent hoarseness, but laryngeal involvement of the BP could be excluded.

After two weeks of worsening, we added oral mycophenolate mofetil (MMF) at a dose of 625 mg/m^2^ twice daily (MMF local dosing regimen, note that recommended standard dose in children is 600 mg/m^2^ twice daily). Within days, the patient’s skin improved and the number of new lesions decreased. We interpreted this improvement as delayed response to dapsone rather than response to MMF, which usually takes several weeks to set in. Over the following weeks, we slowly weaned the patient off systemic corticosteroids and then reduced the MMF dose in two steps over two months. After another two months of clinical remission, we also stopped treatment with dapsone. After 12 months the patient was off all medication. Anti-BP180 antibody values significantly decreased over the course of three months, parallel to clinical improvement. Also, the number of leukocytes (including eosinophils) and thrombocytes decreased and normalized. At the time of submission, the patient had been free of symptoms for two years. Due to parental fear of relapse, the patient had not received any further vaccinations.

### Analysis of all reported infantile BP cases, including own patient cohort

#### Clinical characteristics

The literature review of all obtainable reports between the years 1977 and 2013 including our own cases revealed 53 reports [8,11-14,22-68] with a total of 81 cases of BP occurring in children within the first year of life but beyond the neonatal period (Additional file 1: Table S1). While very few cases were reported before the year 2000, there has been a significant increase since then (Additional file 2: Figure S1). The mean and median age was approximately four months with 64% of cases between three to five months. The gender ratio male to female was 39 to 38. In four cases gender was not stated. Moderately severe and severe (generalized) disease (>10% body surface area – BSA) was seen in 83.9% of cases (n = 68 of 81). All children showed at least some involvement of the hands and feet. Mucosal blistering was present in 14.8% of cases (n = 12 of 81); four of these patients had severe disease (Table 2).

**Table 2:** **Clinical characteristics of all reported infantile BP cases, including own patient cohort**

**No of cases**	**N = 81**	**Comments**
**Mean (median) age/age range**	4.5 (4) months/1–12 months	
**Gender M/F**	39/38 (4 unknown)	
**Extent of skin in involvement**		
• Localized/mild disease (+/− few disseminated plaques)	N = 10 (12.3%)	
• Generalized/moderately severe and severe disease	N = 68 (83.9%)	
• N/A	N = 3 (3.7%)	
• Involvement of hands and feet	N = 81 (100%)	
**Involvement of oral mucosa (with generalized disease)**	N = 12 (14.8%)	All children with oral lesions had generalized skin involvement
		Severe disease N = 5
**No of children vaccinated prior to onset**	N = 25 (30.8%)	Latency between vaccination and onset of disease: 1 day - 4 weeks
• DPTP +/− others,	N = 22	
• Rotavirus,	N = 2	
• DPTP plus Rotavirus	N = 1	
**No of patients with a relapse**	N = 12 (14.8%)	
**Outcome**		
• Cured	N = 76 (93.8%)	Patient had congenital immune deficiency
• In remission under treatment at time of report	N = 3 (3.7%)	
• Still symptomatic at time of report	N = 1 (1.2%)	
• Death	N = 1 (1.2%)	

**N/A**: Not Available; **DPTP**: Diphteria, Pertussis, Tetanus, Poliovirus.

98% (n = 79 of 81) of children affected had previously been healthy. One patient had a congenital T-cell lymphocytopenia and one child had been diagnosed with Hyper IgE-syndrome. The general condition at the time of presentation was good in the majority of cases; some patients were irritable, likely due to pruritus. However, one child with a very delayed initiation of appropriate treatment presented with significant morbidity, including severe weight loss, dehydration and failure to thrive, as well as developmental delay [41]. One of our own patients was also severely affected during a relapse where he refused oral intake and lost weight (see index case above). Both children improved quickly once sufficient treatment was established.

Twenty five children (30.8%) had been vaccinated within days or weeks prior to the onset of disease, the majority with the standard mix of passive vaccines recommended in this age group. Two of our five own cases had received a newly recommended oral vaccine against Rotavirus prior to the onset of disease. This has not been reported before. In two children a febrile infection was reported prior to the onset of disease [37] or prior to a relapse [27], this report.

### Pathophysiology and diagnostic features

Histology, if reported, showed dermal edema, an inflammatory infiltrate dominated by eosinophils and subepidermal blistering. DIF showed IgG and/or C3 along the basement membrane in 72 cases (90%), in 12 cases (15%) there were additional IgA deposits, in four cases there were IgM- and in one case IgE-deposits. In immunoblot analyses reported in 20 patients, 15 sera recognized a 180 kDa protein, five sera recognized a 230 kDa protein, and one serum both.

**Figure 2:**
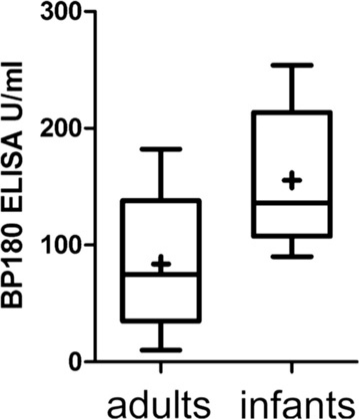
**ELISA values in infantile and adult BP.** Anti-BP180 ELISA values in our infantile BP cohort were significantly higher, compared to a control group of newly diagnosed adult BP patients (normal value <9 U/ml; boxplot analysis; whiskers: minimum and maximum values; bottom and top of boxes: first and third quartiles; band inside box: median; cross: mean).

ELISA values were reported in only 21 (25.9%) cases. All of these patients had antibodies against the NC16A-domain of BP180; two also had additional anti-BP 230 antibodies. Comparison of ELISA values of reported cases from different centers is not fully possible because of different commercial and non-commercial ELISA systems used. In our own cohort, BP180 NC16A ELISA values in infantile patients were significantly higher than in a control group of 28 adults newly diagnosed with BP in our center in the same time period (Figure 2). Extremely high values in our cohort and in reported patients seemed to be associated with more extensive disease and the need for systemic treatment.

A blood cell count was reported in 37 patients, the mean white blood cell count was 23.9 G/l (range <10-120G/l, median 19.4). The percentage of eosinophils had a mean of 23% (range 7-66%, median 19%).

### Treatment modalities

The majority of patients were treated with systemic corticosteroids (50.6%) with or without additional erythromycin or other antibiotics. 19.8% of patients were treated with a combination of systemic corticosteroids and dapsone or sulfapyridine, and 9.9% were treated with topical corticosteroids alone. 13.7% of patients (n = 11) needed a combination of multiple agents (Table 3). All but one patient reached remission eventually. However, the patient with concomitant congenital T-cell lymphocytopenia died from unknown cause three months after having received two doses of rituximab for severe disease. Relapses were not common (14.8%, n = 12) (Table 3 and Additional file 3: Table S2).

**Table 3:** **Treatment Modalities of Infantile BP Patients**

**Treatment**	**No of cases (% of total N = 81)**	**Comments**
Topical corticosteroids alone	N = 8 (9.9%)	Good response
Topical corticosteroids + IVIG	N = 1 (1.2%)	Several relapses for one year
Topical corticosteroids + erythromycin	N = 1 (1.2%)	Good response
Systemic +/− topical corticosteroids (+/− antibiotics)	N = 41 (50.6%)	Good response
Systemic corticosteroids + dapsone/ sulphapyridin (+/− antibiotics)	N = 16 (19.8%)	Good response
Dapsone/ sulphapyridin alone	N = 2 (2.5%)	One relapse under treatment. Same treatment was attempted in one other patient without success, so steroids were added.
No treatment	N = 1	
N/A	N = 1	
Corticosteroids +/− dapsone plus other medications due to poor response	N = 11 (13.7%)	
• Azathioprine	N = 1	No response
• Cyclosporine	N = 2	Good response in N = 1 Partial response in N = 1
• Mycophenolate mofetil	N = 7	Moderate response in N = 7
• Erythromycin and nicotinamide	N = 8	Good response in N = 3 Partial / uncertain response in N = 5
• IVIG	N = 8	Good response in N = 2 Partial/ uncertain response in N = 6
• Rituximab	N = 3	Good response N = 2. Partial response N = 1. One sudden death in one of those two patients after three months (child had congenital immune deficiency).
• Omalizumab	N = 1	Good response

**IVIG:** Intravenous immunoglobulins.

## Discussion

Infantile BP is considered very rare. Prospective studies are therefore difficult to perform. Incidence in Israel was estimated to be 2.36:100,000 per year [13]; however, in most countries no central registry exists and the disease might be under-recognized. We present a detailed characterization of a current cohort of five infants with BP from our departments. Furthermore we performed a comprehensive analysis of all cases reported in the literature (age 1–12 months) with respect to clinical and laboratory characteristics and treatment modalities. Taken together the results allow for the following conclusions.

### Diagnostic features

Laboratory test results in infantile BP generally resemble those in adult BP. Linear IgG and/or C3 depositions at the basement membrane in DIF are the diagnostic hallmark. Autoantibody profiles, as detected by various methods, are comparable to those in adults with BP [69]: autoantibodies against the NC16A domain of BP180 are more frequent than anti-BP230 antibodies.

We propose the following minimal diagnostic criteria for infantile BP: typical clinical picture (urticarial plaques and blisters, acral distribution) and linear IgG and/or C3 deposition at the basement membrane in DIF. Further diagnostic pointers are the presence of serum autoantibodies against BP180 and/or BP230. and – even though less specific – subepidermal blistering with an eosinophil rich inflammatory infiltrate in conventional histology.

Even though ELISA results were only reported in a minority of cases, and different test systems used do not allow for direct comparison, the reported autoantibody levels in infants seem fairly high. Comparing ELISA values of our five infants with a control group of 28 adults newly diagnosed with BP in our center in the same time period, we found that the mean and median levels of anti-BP180 NC16A antibody levels in infants were significantly higher. These ELISA values had been measured with the same test system (see Methods).

The clinical relevance of antibody testing in infantile BP has been contested [14]. Nevertheless – when tested – patients with a more recalcitrant disease course demonstrated high autoantibody levels. In our cohort, higher values at presentation correlated with the need for more aggressive and longer-term treatment, and values increased before relapses. Therefore, it appears reasonable to take into account the levels of BP180-specific autoantibodies in infantile BP when making treatment decisions.

### Patient characteristics/clinical features

At disease onset, the mean age of children was around four months. As opposed to previous reports [13], there was no significant female predominance.

No common trigger was identified. A large number of patients had either been vaccinated or suffered an infection prior to the onset or relapse of disease (Table 2, Additional file 1: Table S1 and Additional file 3: Table S2). The type of infection or vaccine varied. It can be speculated that a modulation of the immune system might play a role in triggering or unmasking an underlying subclinical BP. Nevertheless, especially due to the high number of infants receiving vaccination, this association might be purely coincidental and we believe that the term postvaccination infantile BP should be used with caution.

Cases of adult BP associated with malignancy exist, even though the causal relation remains unclear. In contrast, no case of infantile BP in relation with a malignant neoplasm has been reported. Furthermore, unlike in adult BP [70,71], drugs do not seem to play a major role in triggering infantile BP.

Within the age group of four weeks to 12 months, the clinical picture was moderately severe to severe (generalized) in over 80% of cases. Acral blistering was present in all children, while mucosal involvement was uncommon. In localized disease, hands and feet were usually affected. There was no case of isolated genital infantile BP. Taken together, involvement of the hands and feet can be considered as a clinical hallmark and diagnostic clue of infantile BP. This is in contrast to childhood and adult BP [1,4,69]. Important differential diagnoses of infantile BP are listed in Table 4.

**Table 4:** **Important Differential Diagnoses of Infantile BP**

**Autoimmune blistering skin diseases**	• Linear IgA dermatosis
	• Epidermolysis bullosa acquisita
**Hereditary**	• Epidermolysis bullosa
	• Porphyria
**Infectious**	• Bullous impetigo
**Others**	• Pompholyx
	• Bullous mastocytosis
	• Insect bites
	• Insect bite like reaction of hematologic malignancy

Most infants were doing well at the time of presentation despite some irritability, likely due to pruritus. However, individual children with significant morbidity including difficulty breathing and feeding, and weight loss, have been reported.

Even though initial presentation is often severe, the prognosis of infantile BP is excellent, with all but one patient reaching complete remission. That child had only been followed up short-term at the time of publication [8] and subsequent remission is possible. One infant passed away shortly after having been discharged from hospital. This child had received several doses of rituximab and had an underlying immune deficiency, which might have played a role.

The number of relapses was low. It seems that relapses can be triggered by infections or that they occurred in patients where tapering of corticosteroids was started early. Also, relapses were more frequent in patients who did not receive systemic corticosteroids (Additional file 3: Table S2). Once the disease has been controlled for several months, the likelihood of a relapse is extremely small.

### Treatment algorithm

**Figure 3:**
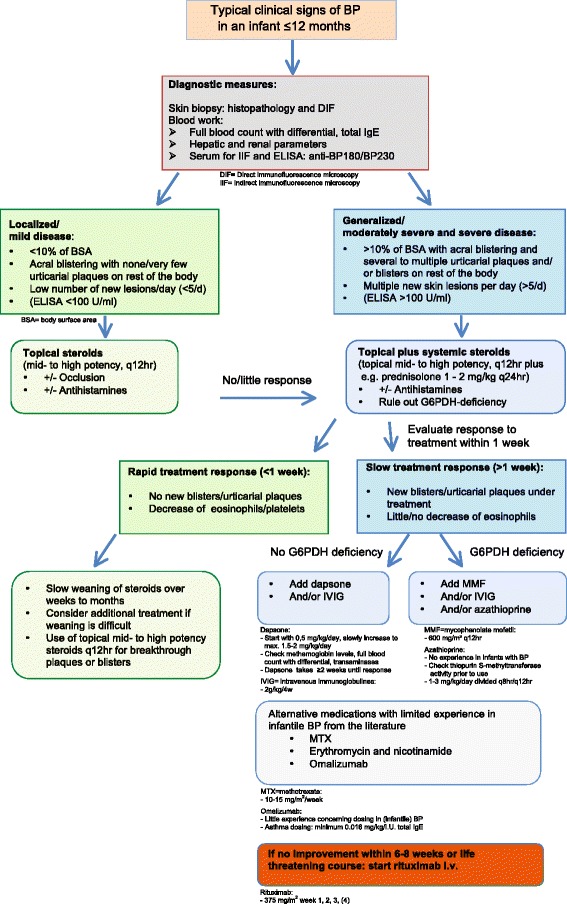
**Step-by-step diagnostic and treatment algorithm.** The algorithm was developed taking into account disease severity, response to initial treatment and specific aspects of steroid sparing agents.

In contrast to adult BP, no treatment guidelines for infantile BP exist [15-18,72], and there has been little discussion on possible criteria for choosing the right treatment. After a comprehensive analysis of reported treatments in all published cases of infantile BP – together with lessons learned from our own cohort – we propose a first treatment algorithm. This step-by-step diagnostic and treatment algorithm takes into account disease severity, response to initial treatment and specific practical aspects of steroid sparing agents. It is based on general experience with the different medications in infants and the treatment recommendations published for adult BP (Figure 3).

After the diagnosis is established, all patients should receive treatment with mid- to high-potency topical corticosteroids. Children with moderately severe or severe disease (generalized, >10% BSA) usually require additional treatment with systemic corticosteroids. If the treatment response is slow or high doses of corticosteroids are needed for disease control, additional steroid sparing agents should be considered. Dapsone seems to be the agent of choice as it is usually well tolerated, effective, and is frequently used for other autoimmune blistering diseases of infancy and childhood, such as linear IgA dermatosis. Other steroid sparing agents used are IVIGs and MMF. Little or no experience exists for erythromycin-, methotrexate-, cyclophosphamide or azathioprine treatment in infants with BP. Rituximab is to be reserved as rescue treatment for the most severe cases [49,67]. The full potential and dosing of omalizumab in infantile BP warrant further investigation [56,73].

After clinical remission for several months, treatment discontinuation can be considered. In our experience ELISA autoantibody values can take a long time to normalize and are therefore not always helpful for deciding when to end treatment.

## Conclusions

Infantile BP is considered a rare disorder; however an increasing number of reports during the last years show that it might have been under-recognized. As the disorder is not well known to general pediatricians and dermatologists, most infants are not promptly diagnosed and undergo multiple examinations before establishment of the correct diagnosis.

Infantile BP presents with urticarial plaques and blisters. Involvement of hands and feet is present in all cases. The clinical picture of infantile BP is characteristic. It is therefore a realistic aim to make the diagnosis early, avoid unnecessary diagnostic measures, and treat appropriately to avoid severe morbidity.

Pathogenesis and diagnostic criteria are comparable to adult BP, yet ELISA levels seem to be higher in infants. The overall disease outcome is favorable. Based on the results of this study we have established a first step-by-step diagnostic and treatment algorithm, taking into account disease severity, response to initial treatment and specific aspects of steroid sparing agents.

## Additional files

Additional file 1: Table S1. All cases of infantile BP in the literature and this study. Additional file 2: Figure S1. The number of published infantile BP cases has significantly increased since 2000. Additional file 3: Table S2. Relapses of infantile BP.

## Abbreviations

BP: Bullous pemphigoid
BP180: Bullous pemphigoid 180 kDa antigen/collagen XVII
BP230: Bullous pemphigoid 230 kDa antigen
BSA: Body surface area
DIF: Direct immunofluorescence microscopy
IIF: Indirect immunofluorescence microscopy
IVIG: Intravenous immunoglobulins
MMF: Mycophenolate mofetil
