# Supplementary material for: Bullous pemphigoid in infants: characteristics, diagnosis and treatment
Source: Orphanet J Rare Dis. 2014 Dec 10;9:185. doi: 10.1186/s13023-014-0185-6 (PMC4302581; doi:10.1186/s13023-014-0185-6)
Supplement: Additional file 1: Table S1. — All cases of infantile BP in the literature and this study. [file 13023_2014_185_MOESM1_ESM.docx]

**Additional Table 1) All Cases of Infantile BP in the Literature and this Study**

|  | **Author / Year**  **(No of reported**  **patients)** | **Age  (months) /**  **Gender** | **Extent of disease**  **Hands/Feet (HF)** | **OM** | **DIF /**  **IIF /**  **Immunoblot** | **ELISA** | **WBC**  **(% Eosinophiles)**  **Thrombocytes** | **Treatment** | **Time Until Remission**  **Relapse**  **Duration of Treatment** | **Special Aspects** |
| --- | --- | --- | --- | --- | --- | --- | --- | --- | --- | --- |
| 1 | Gould 1977  (1) | 3/? | Disseminated  HF+ | - | DIF: IgG  IIF: + | N/A | N/A | Prednisone 20 mg/d  plus topical betamethasone | Days  No relapsee |  |
| 2 | Marsden 1979  (1) | 3/M | Initially localized,  then generalized  HF+ | - | DIF: IgG,C3  IIF: 1:80 | N/A | N/A | Sulphapyridin 250 mg | Improvement within 10 days  One relapse |  |
| 3 | Marsden 1980  (2) | 8/F | N/A  HF+ | N/A | DIF: IgG, C3  IIF: N/A | N/A | N/A | Sulphapyridin |  | One case of two in  this paper had been  reported in 1979 paper |
| 4 | Hernandez  -Aguado 1982  (1) | 5/F | Generalized  HF+ | - | N/A | N/A | 13.5 (8) | Prednisone 2.5-1 mg/kg/d |  |  |
| 5 | Marsden 1983  (1) | 4/F | Generalized  HF+ | - | DIF: IgG, C3  IIF: 1:1280  IB: 230kD  Maternal serum  negative | N/A | 60.3 x10^9^/l (65) | a)Sulfapyridine 250🡪 500 mg/d  b)Systemic prednisolone 20mg/d | Little response to sulfapyridine  Improvement 24 h post steroid  Remission slow  Tapering over 3 years | Same case as in  Edwards 1998 |
| 6 | Zebede 1987  (1) | 3/M | Initially localized,  then generalized  HF+ | - | DIF: IgG, C3  IIF: + | N/A | N/A | N/A | N/A |  |
| 7 | Tani 1988  (1) | 4/F | Gzneralized  HF+ | **+** | DIF: IgG, IgA, C3  IIF: - | N/A | 24.4 x10^9^/l (45) | a) Dapsone 20 mg/d  b) Systemic betamethasone  0.7 mg/kg 🡪1.4 mg/kg | Remission 6 days after  higher steroid dose was started |  |
| 8 | Nemeth 1991  (1) | 2.5F | Mainly localized,  few disseminated  lesions  HF+ | **+** | DIF: IgG, C3  Negative IIF: | N/A | 12.3 x10^9^/l (9%)  Tc 400x10^9^/L | a) Prednisone 1 mg/kg  plus topical hydrocortisone | Improvement within 3 d,  Remission after 3 weeks | Mother’s IIF: neg |
| 9 | Oranje 1991  (1) | 4/M* | Generalized  HF+ | - | DIF: IgG, C3,  (IgA,IgM, IgE)  IIF: - | N/A | 29% Eosinophils | Prednisolone 2mg/kg/d | Rapid response,  6 months of treatment | Vaccine DPTP  “shortly” before |
| 10 | Ostlere 1993  (1) | 12/M | Generalized  HF+ | N/A | DIF: IgG, C3  IIF: –  IB: 190kD | N/A | 20 x10^9^/l | a) Topical clobetasol diproprionate  b) Prednisolone 1 mg/kg/d  c) Sulphapyridine 500 mg  BID🡪 TID  d) Flucloxacillin | Initially good response to  topical treatment  Relapse during fever (39°C),  then need for systemic treatment |  |
| 11 | Kirtschig 1994  (1) | 6/F | Generalized  HF+ | - | DIF: IgG, C3  IIF: IgG | N/A | N/A | Prednisolone 30mg/d  plus sulphapyridine 250 mg/d | Rapid response  Slow remission | 3 years of treatment |
|  | **Author / Year**  **(No of reported**  **patients)** | **Age  (months) /**  **Gender** | **Extent of disease**  **Hands/Feet (HF)** | **OM** | **DIF /**  **IIF /**  **Immunoblot** | **ELISA** | **WBC**  **(% Eosinophiles)**  **Thrombocytes** | **Treatment** | **Time Until Remission**  **Relapse**  **Duration of Treatment** | **Special Aspects** |
| 12 | Cambazard 1994  (1) | 4/M* | Generalized,  rapid spread  HF+ | **+** | DIF: IgG, C3  IIF: IgG (1:100)  IB: 230kD (1:200) | N/A | 10.1 x10^9^/l (N/A) | Prednisolone 2mg/kg/d | Rapid response  6 months of treatment | Tetracoque (DPTP)  After revaccination  development of a  transient urticarial rash. |
| 13 | Nagano 1994  (1) | 3/M | Generalized  HF+ | - | DIF: IgG, C3  IIF: IgG 1:512  IB: 180kD | N/A | N/A | a) Erythromycin 250mg/d  b) Dapsone 20mg/d  and prednisolone  9 mg/d 🡪14 mg/d 🡪18 mg/d | Improvement with high dose of  prednisone within 2weeks  Remission 3 weeks after dapsone  and an increased dose of prednisolone | No maternal antibodies  in serum or breast milk. |
| 14 | Wisuthsarewong  1997 (1) | 7/F | Generalized  HF+ | - | N/A | N/A | N/A | High dose systemic steroids |  |  |
| 15 | Edwards 1998  (1) | 4/F | Generalized  HF+ | N/A | DIF:IgG,C3  IIF:1:320 IgG  IB: 230 kDa | N/A | N/A | Prednisolone 20 mg/d  plus sulfapyridine 250 mg/d | Complete remission after 3 years  No relapse | Multiple problems from  long-term steroids |
| 16 | Cunha 1998  (1) | 2.5/M*§ | Generalized  HF+ | N/A | DIF: C3  IIF: + 1:40  IB: 180, 230 kD | N/A | 8.8 x10^9^/l (6)  Initially, later  34.600 (34)  IgE = 248.7 U/ml | a) Prednisone, 2 mg/kg/day,  b) Dapsone, 2 mg/kg/day,  c) Erythromycin, 26 mg/kg/day  plus nicotinamide, 5 mg/day. | Response within 1 month  Remission within 3 months  Until complete remission pt. still  had high WBC, eosinophils and  platelets | DPT vaccine, SABIN  vaccine, Vaxgrip  (influenca) 5 days prior. |
| 17 | Amos 1998  (1) | 3/F * | Localized  HF+ | - | Negative (?) | N/A | Normal (8) | Amcinonide ointment | 2 months |  |
| 18 | Trueb 1999  (1) | 6/? | Generalized  HF+ | **+** | DIF: IgG, C3  IIF: IgG (1:10) | N/A | 40 x10^9^/l (33) | a) Dapsone (5 mg/kg/day),  b) Dexamethasone (3 mg/day)  c) IVIG (5 g/day) x5d  d) Oral prednisone (2 mg/kg/day).  e) Cyclosporine 1🡪5 mg/kg  f) Dexamethasone (3 mg/day) | Rapid improvement after IVIG  and steroids  Relapse after 2 weeks,  improvement with Cyclosporine,  then sudden deterioration dyspnea with blistering involving the upper respiratory tract. |  |
| 19 | Chimanovitch  2000  (2) | 4/F | Generalized  HF+ | - | DIF: IgG, C3  IIF: IgG | N/A | N/A | Methylprednisolone (0.5/kg/d)  Dapsone (1 mg/kg/d)  +vitamin E (600 U/d) | Response within 4 days,  Remission within 4 weeks  Tapering of steroids over  4 months  Dapsone for >7 months  No relapse |  |
| 20 | Chimanovitch  2000 | 5/M | Generalized  HF+ | - | DIF: C3 IgG,  (IgA, IgM)  IIF: + (IgG) | N/A | N/A | Prednisolone (0.5mg/kg/d)  Dapsone +vitamin E | Response withing days  Remission within 2 months  Treatment duration 3 months  No relapse |  |
|  | **Author / Year**  **(No of reported**  **patients)** | **Age  (months) /**  **Gender** | **Extent of disease**  **Hands/Feet (HF)** | **OM** | **DIF /**  **IIF /**  **Immunoblot** | **ELISA** | **WBC**  **(% Eosinophiles)**  **Thrombocytes** | **Treatment** | **Time Until Remission**  **Relapse**  **Duration of Treatment** | **Special Aspects** |
| 21 | Segurado 2000  (2) | 8/F | Generalized  HF+ | - | DIF: C3 IgG  IIF: - | N/A | Incr. WBC and  eosinophilia | Prednisone 2mg/kg/d  Sulfapyridin | Remission within 2-3 weeks,  Treatment duration 2 months  No relapse | Respiratory tract infection |
| 22 | Segurado2000 | 4/M* | Generalized  HF+ | - | DIF: C3 IgG  IIF: N/A | N/A | N/A | Prednisone 2mg/kg/d | Remission within 2-3 weeks,  No relapse  Treatment duration 3 mo | DPTP 24h prior |
| 23 | Baykal 2001  (1) | 3,5/M* | Generalized  (rapid spread)  HF+ | - | DIF: IgG, C3  IIF: IgG, 1:10 | N/A | 21 x10^9^/l (N/A)  Tc 650 x10^9^/l | Methylprednisolone 5 mg  (1mg/kg per day) | Remission after 2weeks | 24h after vaccine  (DHFIT) |
| 24 | Petronius 2002  (2) | 4/ F | Generalized  HF+ | - | DIF: IgG, C3, (IgA)  IIF: + 1:10 | N/A | 18 x10^9^/l (18) | 1) Methylprednisolone 3 mg/kg/d,  during relapse topical clobetasole | Remission after 3 weeks  Rapid tapering over 7 weeks  Relapse on palms and soles |  |
| 25 | Petronius 2002 | 3.5/F* | Generalized  HF+ | - | DIF: IgG, C3  IIF: - | N/A | 22.4 x10^9^/l (normal) | 2) Methylprednisolone 0.5-0.75 mg/kg/d,  dapsone (max 4 mg/kg/d) | Only moderate response to steroids  Good reponse after 4mg/kg dapsone.  No relapse  Treatment duration 4-6 months. | Vaccinations and  oral vitamins 4w prior |
| 26 | Fisler 2003  (2) | 5/M* | Localized  HF+ | - | DIF: IgG, C3, (IgA)  IIF: ND | N/A | N/A | Topical steroids under occlusion  for 2 weeks | Remission within several weeks,  Relapse when stopping treatment | Vaccination 5days prior |
| 27 | Fisler 2003 | 8/ F | Generalized  HF+ | **+** | DIF: IgG, C3  IIF: ND | N/A | N/A | a)Prednisone 2mg/kg/d  + dapsone 🡪 not tolerated  b)Erythromycin + nicotinamide for  3 weeks w/o response  c) Mycophenolate mofetil 20 mg/kg/d,  after 1 mo reduction of Prednisolone  to 1 mg/kg/d | No complete remission  at time of report |  |
| 28 | Singalavanija  2003 (4) | 2/F | Generalized  HF+ | **+** | DIF: IgG  IIF: - | N/A | N/A | a)Prednisone | Rapid response |  |
| 29 | Singalavanija  2003 | 3/M | Generalized  HF+ | **+** | DIF: IgG, C3  IIF: + | N/A | N/A | a)Prednisone | Rapid response |  |
| 30 | Singalavanija  2003 | 6/M | Generalized  HF+ | + | DIF: IgG, C3  IIF: + | N/A | N/A | a)Prednisone | Slow response |  |
| 31 | Singalavanija  2003 | 8/F | Localized,  then generalized | + | DIF: IgG, C3  IIF: N/A | N/A | N/A | a)Prednisone + dapsone  b) Mycophenolate mofetil | Slow response |  |
| 32 | Kuenzli 2004  (1) | 7/F | Generalized  HF+ | N/A | DIF: IgG, C3, (IgA)  IIF: IgG  IB: 180kDa | N/A | Thrombocytes  very high  🡪 aspirin | a) Prednisone 1 mg/kg/d  b)Sulfapyridine 90 mg BID | Remission within 2 weeks.  Relapse during tapering of steroids.  Treatment duration 3 months. | Dramatic weight loss  w/o steroids/ treatment  (Homöopathic meds) |
|  | **Author / Year**  **(No of reported**  **patients)** | **Age  (months) /**  **Gender** | **Extent of disease**  **Hands/Feet (HF)** | **OM** | **DIF /**  **IIF /**  **Immunoblot** | **ELISA** | **WBC**  **(% Eosinophiles)**  **Thrombocytes** | **Treatment** | **Time Until Remission**  **Relapse**  **Duration of Treatment** | **Special Aspects** |
| 33 | Sousa et al 2005  (1) | 6/M | Generalized  HF+ | N/A | DIF: IgG, C3  IIF:+ | N/A | 20.4 x10^9^/l (18) | Deflazacort 2mg/kg/d,  BMV topically | Rapid resolution. |  |
| 34 | Merida 2005  (1) | 3/F* | Initially localized,  After 2^nd^ vaccine  generalized | - | DIF: IgG, C3 | N/A | N/A | Deflazacort 1mg/kg/d | Improvement within 2 weeks,  Treatment duration 3 months  5 years of f/u | DPTP, HepB, HIB  2 weeks prior and  3 days before worsening |
| 35 | Voltan 2005  (3) | 4/M | Generalized  HF+ | - | DIF: IgG, C3  IIF: + 1:20 | N/A | N/A | Oral betamethasone | Remission within 2 weeks.  Treatment duration 4 months |  |
| 36 | Voltan 2005 | 2/F | Generalized  HF+ | - | DIF: IgG, C3  IIF:+ 1:40 | N/A | N/A | Prednisolone 2mg/kg/d for 3w  Oral Betamethasone for 2 w | Good response to betamethasone.  Treatment duration 6 months |  |
| 37 | Voltan 2005 | 3/F | Generalized  HF+ | - | DIF: IgG, C3, (IgA)  IIF: - | N/A | 19.4 x10^9^/l (34) | Erythromycin 50mg/kg/d 10 days  Oral Betamethasone (dose equivalent  1 mg/kg/d🡪2 mg/kg/d)  Response to higher dose | Moderately rapid response,  Treatment duration several month  after remission |  |
| 38 | Chiaverini 2006  (4) | 2/M | Generalized  HF+ | - | DIF: IgG, C3  IIF: IgG1+  IB: 180kD | Anti BP180  192U/ml  (norm<9,  MBL, Nagoya) | 27.3 x10^9^/l (32) | Prednisone 2 mg/kg/d, BMV OD | Rapid response.  Treatment duration 6 months |  |
| 39 | Chiaverini 2006 | 2/F | Generalized  HF+ | - | DIF: IgG, C3  IIF: IgG1+  IB: 180kD | Anti BP180  187 U/ml  (norm<9,  MBL, Nagoya)) | 18.1 x10^9^/l (37) | Prednisone 2 mg/kg/d 🡪3 mg/kg/d,  BMV OD | Slow response.  Treatment duration 6 months |  |
| 40 | Chiaverini 2006 | 4.5/F* | Generalized  HF+ | - | DIF: IgG, C3  IIF: IgG1+  IB: 180kD | Anti BP180  226 U/ml  (norm<9,  MBL, Nagoya)) | 22.4 x10^9^/l (48) | Prednisone 2 mg/kg/d, BMV OD | Rapid remission,  Treatment duration 6 months. |  |
| 41 | Chiaverini 2006 | 3/ F | Generalized  HF+ | - | DIF: IgG, C3  IIF: IgG1+  IB: 180kD | Anti BP180  139 U/ml  (norm<9,  MBL, Nagoya)) | 12.6 x10^9^/l (9) | Betamethasone diproprionate OD | Rapid remission,  Treatment duration 4 weeks | DPT vaccine 48 hrs  before |
| 42 | Wang 2006 (1) | 6/M | - | - | N/A | N/A | N/A | Steroids |  |  |
| 43 | Schmidt 2007 (1) | 6/M | Generalized | + | N/A | Anti BP180  3528 U/ml  (norm<9,  MBL, Nagoya) | N/A | a) Prednisolone pulses (3x150 mg/pulse)  b) Prednisolone 7.5mg/d  c) Dapsone 22 mg/d  d) Mycophenolate mofetil 320mg/d  e) Cyclophosphamide (500 mg/m² /mo)  f) IVIG  g) Rituximab 375mg/m², w 1,2,3,4,  four additional doses 8-10 mo later | Little to no response to several  immunosuppressants.  No response to rituximab in the  first 6 months.  Partial remission after 12 months  Treatment duration> 26 months. |  |
| 44 | Sugawara  2007 (1) | 3/M | Generalized  HF+ | - | DIF: IgG, C3  IIF: IgG 1:80 | Anti BP180  187.29 U/ml  (norm <15) | 8 x10^9^/l (18.2) | a) Prednisolone (10🡪20 mg/d)  b) Erythromycin (21 mg/d)  c) Dapsone 12.5 m/d  d) Dexamethasone 3mg/d (d17)  e) Methylprednisolone 80 mg/d (d57)  b) IVIG 2.5g/d (300mg/kg/d) x2 | More than 2 months until remission  Two relapses with rapid response  to IVIG |  |
|  | **Author / Year**  **(No of reported**  **patients)** | **Age  (months) /**  **Gender** | **Extent of disease**  **Hands/Feet (HF)** | **OM** | **DIF /**  **IIF /**  **Immunoblot** | **ELISA** | **WBC**  **(% Eosinophiles)**  **Thrombocytes** | **Treatment** | **Time Until Remission**  **Relapse**  **Duration of Treatment** | **Special Aspects** |
| 45 | Xiao 2007  (1) | 3,5/M* | Generalized  HF+ | N/A | DIF: C3  IIF: IgG | Anti BP180  57 U/ml | 13 x10^9^/l (27)  IgE 2910 IU/ml | IVIG 4x 0.4g/gl  over 1 w (total 20g)  + topical corticosteroids | Several relapses  Remission after 1 year |  |
| 46 | Martinez-  DePablo 2007  (4) | 5/M | Generalized  HF+ | N/A | DIF: IgG, C3,(IgA)  IIF: 1:20 | Anti BP180  IgG 33,1U/ml  IgA 86,7 U/ml | N/A | Prednisolone (2mg/kg/d) | Quick response within days |  |
| 47 | Martinez  -DePablo 2007 | 5/M | Generalized  HF+ | N/A | DIF: IgG, C3, (IgA)  IIF: 1:10 | N/A | ↑WBC (14) | a) Topical mometasone for 1w  b) Prednisolone (1 mg/kg/d) | No response to topical steroids.  Remission 2 weeks after systemic  Steroids.  Treatment duration: 3 months |  |
| 48 | Martinez  -DePablo 2007 | 12/M | Generalized  HF+ | N/A | DIF: IgG, C3 | N/A | N/A | a) Dapsone  b) Prednisolone (1 mg/kg/d) | Remission within 1 month,  Treatment duration: couple months |  |
| 49 | Martinez  -DePablo 2007 | 4/M | Generalized  HF+ | N/A | DIF: IgG, C3 | Anti BP180  IgG 52,5 U/ml  IgA 49,7 U/ml | N/A | Prednisolone (1 mg/kg/d) | Remission within 2 weeks  Treatment duration: 4 months |  |
| 50 | Santos 2007 (1) | 5/M | Localized | - | DIF: IgG, C3 | N/A | 15.6 x10^9^/l (17.2) | a) Topical corticosteroids  b) Oral deflazacort 1.5mg/kg/d | Rapid remission  Relapse after 2 weeks of topical  treatment  Treatment duration: 4 weeks |  |
| 51 | Waisbourd-  Zinman 2008  (7) | 3/F | Localized then  Generalized  HF+ | - | DIF: IgG, C3,IgM  IIF: IgG blister roof | N/A | 120 x10^9^/l (66)  after initiation  of treatment | Prednisone 1mg/kg/d🡪 2 mg/kg/d | Remission within 2 months |  |
| 52 | Waisbourd-  Zinman 2008 | 3/M | Localized,  HF+ | - | DIF: IgG, C3 | N/A | N/A | Systemic prednisone | Rapid reponse |  |
| 53 | Waisbourd-  Zinman 2008 | 3/F | Localized  HF+ | - | DIF: IgG, C3 | N/A | N/A | Systemic prednisolone | Rapid response |  |
| 54 | Waisbourd-  Zinman 2008 | 8/F | Localized  HF+ | - | DIF: IgG, C3 | N/A | N/A | Systemic prednisolone | Rapid |  |
| 55 | Waisbourd-  Zinman 2008 | 4/M | Extensive  HF+ | - | DIF: IgG, C3  IIF: 1:40 | N/A | N/A | Systemic prednisolone | Rapid response |  |
| 56 | Waisbourd-  Zinman 2008 | 4/M | Localized,  HF+ | - | DIF: IgG, C3 | N/A | N/A | No treatment | Rapid response |  |
| 57 | Waisbourd-  Zinman 2008 | 4/F | Moderate localized,  then generalized  HF+ | - | DIF: IgG, C3  DIF: IgG, C3,  IIF: 1:40 | N/A | N/A | a) Prednisolone  b) Dapsone(4 mg/kg/d)  c) IVIG  d) Mycophenolate mofetil | Slow response  Remission after 4 months |  |
|  | **Author / Year**  **(No of reported**  **patients)** | **Age  (months) /**  **Gender** | **Extent of disease**  **Hands/Feet (HF)** | **OM** | **DIF /**  **IIF /**  **Immunoblot** | **ELISA** | **WBC**  **(% Eosinophiles)**  **Thrombocytes** | **Treatment** | **Time Until Remission**  **Relapse**  **Duration of Treatment** | **Special Aspects** |
| 58 | Schulze 2008  (1) | 5/M | Generalized  HF+ | - | DIF: IgG, C3  IIF: +blister roof | Anti BP180  795 U/ml | N/A | a) Oral prednisone 3.5 mg/kg  b) Dapsone d 7, 2mg/kg w2  c) Mycophenolate mofetil (30 mg/kg) w3  d) Cyclosporine (4.2 mg/kg) w3  e) IVIG 2g/kg q4w  f) Rituximab 375mg/m²  w16, 187.5mg/ m²w21 | Remission after 21 weeks |  |
| 59 | Erbagci 2008  (1) | 6/M | Generalized  HF+ | **+** | N/A | N/A | WBC 18.5 x10^9^/l  Eos 6.5 x10^9^/l (35) | Systemic steroids (1 mg/kg/d)  + oral amoxicillin/ clavulanate | Rapid improvement, weaning of  steroids over 2 months  Relapse 1 month after  withdrawal of steroids | Hyper IgE Syndrome  IgE 18700IU/mL |
| 60 | Purvis 2009  (1) | 1.5/M | Generalized  HF+ | - | DIF: IgG, C3  IIF: 1:100  IB: 230kD | N/A | Eos 1.39 x10^9^/l  Tc 589 x10^9^/l | Oral prednisolone (2 mg/kg/d) | Prednisolone for 3 weeks  Treatment duration 3-4 months | Collodial silver ,  KCl, K2SO4,  and (Mg(H2PO4)2)  p.o. 2 weeks prior |
| 61 | Belhadjali 2009  (1) | 6/ ? | Generalized  HF+ | - | DIF: IgG, C3  IIF: +blister roof  IB: 180kD | N/A | Eos 1.5 x10^9^/l | a) Topical steroids (mid-high potency)  b) Oral erythromycin 50mg/kg | Improvement after 2 weeks of  treatment with erythromycin  plus topical steroids.  Remission after 1 month |  |
| 62 | Toyama 2009  (2) | 5/F* | Generalized  HF+ | - | DIF: IgG, C3, IgM  IIF: +blister roof  IB: IgG and IgA  against NC16A  region of BP180 | Anti BP180  >150 U/ml,  (norm <9) | 16.3 x10^9^/l (18) | Topical HC-17- butyrate  BID 🡪OD | Remission after 2 weeks | DPT vaccine 3 d prior |
| 63 | Toyama 2009 | 5/F* | Localized with only  few disseminated  lesions  HF+ | - | DIF: IgG, C3, IgA  IIF: +blister roof  IB: IgG and IgA  against NC16A  region of BP180 | Anti BP180  107.6 U/ml  (norm <9) | 10.5 x10^9^/l (7.7) | Topical clobetasole BID | Response after 1 week.  Remission after 1 month | BCG vaccine 9d prior |
| 64 | Majmudar 2010  (1) | 2/F* | Generalized  HF+ | - | DIF: IgG, C3  IIF: IgG, 1:20 | N/A | 21.9 x10^9^/l (19)  Tc 1031 x10^9^/l | Topical steroids (BMV 0.1%)  🡪Prednisolone 1 mg/kg 🡪2 mg/kg  🡪Erythromycin 35mg/kg + top. steroids | Remission within 2 weeks  of treatment with erythromycin  Steroid taper over 6 months |  |
|  | **Author / Year**  **(No of reported**  **patients)** | **Age  (months) /**  **Gender** | **Extent of disease**  **Hands/Feet (HF)** | **OM** | **DIF /**  **IIF /**  **Immunoblot** | **ELISA** | **WBC**  **(% Eosinophiles)**  **Thrombocytes** | **Treatment** | **Time Until Remission**  **Relapse**  **Duration of Treatment** | **Special Aspects** |
| 65 | Khaled 2010  (1) | 5/F* | Generalized  HF+ | - | DIF: IgG, C3,  IIF: IgG2, IgG3 | N/A | N/A | Topical clobetasol BID | 2 months  No relapse | Vaccination 4 w prior |
| 66 | Hafiji 2010  (1) | 3/M* | Generalized  HF+ | - | DIF: IgG, C3,  IIF: IgG2, IgG3  IB: 180+230 kD | Anti BP180 + | Eos 5.3 x10^9^/l  Tc 608 x10^9^/l | Systemic prednisolone 2mg/kg | Remission within 4 weeks  No relapse  Treatment duration 5 mo | DPTP, HIB, Pneumoc.  vaccine |
| 67 | Valdivielso  -Ramos 2011  (1) | 3/F* | Generalized  HF+ | N/A | DIF: IgG, C3, | N/A | WBC elevated | Deflazacort 1 mg/kg/d | Treatment duration 3 months | Vaccine (DPT,P,HiB,  Meningococcus,  Pneumococcus) 3 w prior  Worsening after  2^nd^/3^rd^ vaccination |
| 68 | Dufour 2012  (1) | 4/M | Generalized  HF+ | - | DIF: IgG, C3, (IgA)  IIF: IgG2, IgG3 | Anti BP180  87 U/ml  Anti BP 230  43 U/ ml | 11.5 x10^9^/l  IgE 636 kU/L | a) Systemic prednisolone (-3 mg/kg)  b)Methylprednisolone pulses  b) Dapsone 2mg/kg(d 9)  c) Omalizumab (d 17)  (100 mg q 2- weekly for 3 mo,  then monthly for 4 mo) | Remission within 3 ½ weeks  No relapse | Very rapid change of  medications.  At time of improvement,  dapsone had been in place for  for 2 w |
| 69 | Fuertes 2012  (1) | 7/M | Generalized  HF+ | + | DIF: C3  IIF: IgG | Anti BP180  174 U/ml | N/A | a) Prednisolone 1 mg/kg 🡪2 mg/kg  plus topical steroids,  b)IVIG 200mg/kg/dx5d  c)Azathioprine 2.7mg/kg/d  d)Rituximab 375 mg/m² x4  🡪 remission  e) Mycophenolate mofetil  to maintain remission (30mg/kg/d) | Remission within 4 months | Cong. T-cell lymphopenia  Child deceased of unclear  cause 3 mo after last  rituximab dose. |
| 70 | Barreau 2012  (1) | 3/F | Generalized  HF+ | - | DIF: + (IgG, C3)  IIF: - | N/A | N/A | a) Topical Steroids | Remission within 5 weeks | Vaccination 2 w prior  No relapse after 2^nd^  vaccination |
| 71 | Lynch 2013  (1) | 3/F* | Generalized  HF+ | N/A | DIF: C3  IIF - | N/A | Eos 2.3 x10^9^/l | a) BMV 0,1% cream  b) Erythromycine 4x62,5 mg/d  c) Prednisolone 1mg/d | Response within days  1 relapse | Vaccination 12 days prior |
| 72 | Pai 2013  (1) | 4/? | Generalized  HF+ | - | DIF: IgG, C3 | N/A | 59.84 x10^9^/l (35)  Tc 950 x10^9^/l  IgE normal | Systemic prednisolone 5mg/d🡪7.5 mg/d  Dapsone 10 mg OD | Response 1 w  Remission 15d  No relapse |  |
|  | **Author / Year**  **(No of reported**  **patients)** | **Age  (months) /**  **Gender** | **Extent of disease**  **Hands/Feet (HF)** | **OM** | **DIF /**  **IIF /**  **Immunoblot** | **ELISA** | **WBC**  **(% Eosinophiles)**  **Thrombocytes** | **Treatment** | **Time Until Remission**  **Relapse**  **Duration of Treatment** | **Special Aspects** |
| 73 | De la Fuente  2013 (3) | 4/F* | Generalized  HF+ | - | N/A | N/A | N/A | Systemic steroids 1.5 mg/kg/d | No relapse | N/A |
| 74 | De la Fuente  2013 | 5/M* | Generalized  HF+ | - | N/A | N/A | N/A | Systemic steroids | No relapse |  |
| 75 | De la Fuente  2013 | 5/M* | Generalized  HF+ | - | N/A | N/A | N/A | Systemic steroids | No relapse |  |
| 76 | Brazzelli 2013 (1) | 12/M | Localized  (Limbs/Face)  HF+ | - | DIF: IgG  IIF: IgG, IgA | Anti BP180  22 U/ml  Anti BP230  23,4 U/ml | N/A | Methylprednisolone 1mg/kg/d  🡪Oral prednisone 1 mg/kg/d | Remission within 3 months | Clinical signs of  chickenpox 1 mo prior |
| **77** | **This report**  (5) | 3/M° | Generalized  HF+ | **+** | DIF: IgG, C3  IIF: IgG  IB: 180kD | Anti BP180  136 U/ml  (norm<9,  MBL, Nagoya)  At relapse:  >150U/ml  Anti BP230 neg. | 10.4 x10^9^/l (10)  At relapse:  54 x10^9^/l (52)  Tc > 1000 x10^9^/l | a) Prednisolone 2mg/kg/d  b) Dapsone 2mg/kg/d  c) IVIG 1g/kg x3  d) Mycophenolate mofetil (30 mg/kg) | Initially rapid response  with disease control.  Relapse within 2 weeks after diagnosis,  on systemic prednisolone (2mg/kg)  and during respiratory tract infection  Slow response after relapse,  need for multiple medications.  Response to dapsone after 2.5 weeks.  Duration of treatment: 8 months | Family history of atopy +  Rotavirus vaccine  4 weeks prior |
| **78** |  | 3/M | Localized with few  disseminated  lesions  HF+ | - | DIF: IgG, C3  IIF: IgG  IB: 180kD pos. | Anti BP180  90 U/ml  (norm<9,  MBL, Nagoya))  Anti BP230 neg. | 16.1 x10^9^/l (23,4) | Topical mid-potency steroids | Rapid response to topical treatment  within days.  No relapse  Duration of treatment: weeks. |  |
| **79** |  | 4/M*° | Generalized  HF+ | - | DIF: IgG, C3  IIF: IgG  IB: 180kD pos. | Anti BP180  156 U/ml  (norm<9,  MBL, Nagoya)  Anti BP230 neg. | 23.4 x10^9^/l (20) | Prednisolone 2mg/kg/d 🡪 1mg/kg/d  plus dapsone 1.5 mg/kg/d | Remission within 1 week,  treatment for 6 months (dapsone)  under control of ELISA titers | Vaccination 4 weeks prior  (DPTP, HiB, HepB,  Rotavirus) |
| **80** |  | 3/F | Generalized  HF+ | - | DIF: IgG, C3 (BM)  IIF: IgG (BR)  IB: 180kD pos. | Anti BP180  125U/ml  (norm<9,  MBL, Nagoya)  Anti BP230 neg. | 25.1 x10^9^/l (13)  Tc 860 x10^9^/l | a) Prednisolone 2 mg/kg/d 🡪 1 mg/kg/d  b) Betamethasone 0.3 mg/kg/d  c) Dapsone 1🡪 0.5 mg/kg/d |  | Rotavirus vaccine  4 weeks prior  Arterial hypertension  Myocardial hypertrophy  🡪 Propranolol |
| **81** |  | 7/M | Generalized  HF+ | - | DIF: IgG, C3  IIF: IgG  IB: 180kD pos. | Anti BP180  154 U/ml  (norm<9,  MBL, Nagoya)  Anti BP230 neg. | 27.3 x10^9^/l (9)  Tc 599 x10^9^/l | a) Prednisolone 1mg/kg/d 🡪 0.5 mg/kg/d  b) Betamethasone  0.4 mg/kg/d 🡪 0.2 mg/kg/d  c) Dapsone 0.5 mg/kg/d | Rapid response to betamethasone  Full remission after 2 months  No relapse  Treatment ongoing |  |

**HF:** Hands/Feet + present, - not present; **OM:** Involvement of oral mucosa; + present, - not present; **DIF:** Direct immunofluorescence microscpopy; **IIF:** Indirect immunofluorescence microscpopy; **IB**: Immunoblot; **BM:** basement membrane;

**BR:** Blister roof; **WBC** White blood cell count; **Eos**: eosinophil granulocytes; **Tc:** thrombocytes; **DPTP**: Diphteria, Pertussis, Tetanus, Poliovirus; **HiB:** Haemophilus influenzae type b; **HepB:** Hepatitis B. * Vaccination prior to onset;

**°** Rotavirus vaccine; **§** Influenca vaccine, **N/A**: not available. Generalized disease = Moderately severe and severe disease
