# Supplementary figures and images for: Bullous pemphigoid in infants: characteristics, diagnosis and treatment
Source: Orphanet J Rare Dis. 2014 Dec 10;9:185. doi: 10.1186/s13023-014-0185-6 (PMC4302581; doi:10.1186/s13023-014-0185-6)

### Published cases of infantile BP

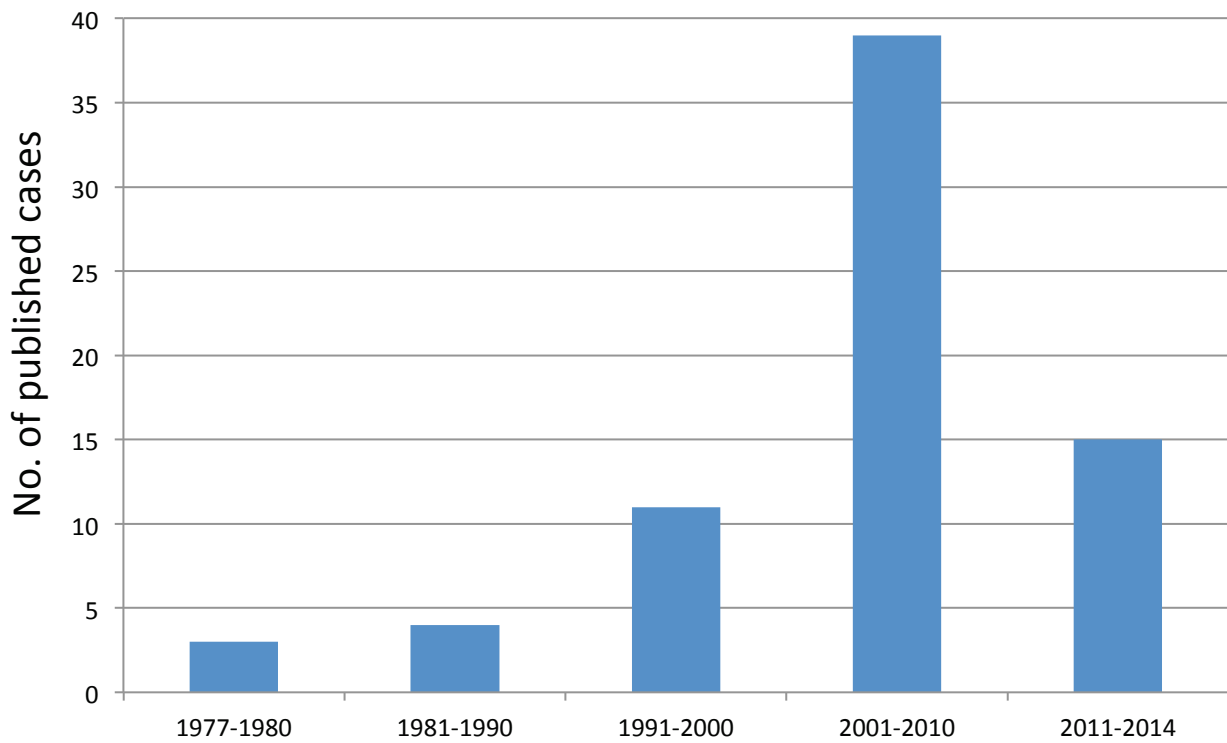

Period of publication

Period of publication

Supplement: Additional file 2: Figure S1. — The number of published infantile BP cases has significantly increased since 2000. [file 13023_2014_185_MOESM2_ESM.pdf]
