# Supplementary material for: Bullous pemphigoid in infants: characteristics, diagnosis and treatment
Source: Orphanet J Rare Dis. 2014 Dec 10;9:185. doi: 10.1186/s13023-014-0185-6 (PMC4302581; doi:10.1186/s13023-014-0185-6)
Supplement: Additional file 3: Table S2. — Relapses of infantile BP. [file 13023_2014_185_MOESM3_ESM.docx]

**Additional Table 2) Relapses of Infantile BP**

| **Publication** | **N=** | **Possible trigger of relapse** | **Treatment at time of relapse** |
| --- | --- | --- | --- |
| Marsden, 1979 | 1 | No trigger identified. | Sulphapyridin only; |
| Ostlere, 1993 | 1 | Febrile infection prior to relapse. | Topical clobetasol diproprionate; |
| Trueb, 1999 | 1 | No trigger identified. | Systemic corticosteroids, dapsone, IVIG; |
| Petronius, 2002 | 1 | No trigger identified. | Methylprednisolone with tapering over 7 weeks; |
| Fisler, 2003 | 1 | No trigger identified. | Topical corticosteroids with tapering after 2 weeks; |
| Kuenzli, 2004 | 1 | Relapse during tapering of steroids. | Systemic corticosteroids and dapsone |
| Xiao, 2007 | 1 | No trigger identified.  Several relapses. | IVIG and topical corticosteroids; |
| Santos, 2007 | 1 | No trigger identified. | Topical corticosteroids |
| Sugawara, 2007 | 1 | No trigger identified. | Systemic corticosteroids, dapsone and other systemic medications, long time to remission; |
| Erbagci, 2008 | 1 | No trigger identified. | Systemic corticosteroids, relapse after cessation of steroids. |
| Lynch, 2013 | 1 | Relapse during tapering of systemic corticosteroids. | Systemic corticosteroids and erythromycin; |
| Own index case | 1 | Respiratory tract infection. | Systemic corticosteroids. |
| **Total** | 12 |  |  |
